# The Effect of Cranberry Consumption on Blood Pressure: A Systematic Review and Meta‐Analysis of Randomized Controlled Trials

**DOI:** 10.1002/clc.70254

**Published:** 2026-04-20

**Authors:** Leyli Zahra Bahreyni, Mohammad Reza Amini, Leila Sheikhi, Ehsaneh Taheri, Pardis Rahimi, Mahnoush Mehrzad Samarin, Fatemeh Sheikhhossein, Sajjad Etesamnia, Negin Lohrasbi, Azita Hekmatdoost

**Affiliations:** ^1^ Student Research Committee, Department of Clinical Nutrition & Dietetics, National Nutrition & Food Technology Research Institute Shahid Beheshti University of Medical Sciences Tehran Iran; ^2^ Nutrition and Food Security Research Center Isfahan University of Medical Sciences Isfahan Iran; ^3^ Food and Beverage Safety Research Centre Urmia University of Medical Sciences Urmia Iran; ^4^ Liver and Pancreaticobiliary Disease Research Center, Digestive Diseases Research Institute Tehran University of Medical Sciences Tehran Iran; ^5^ Student Research Committee, Department of Clinical Nutrition, School of Nutrition and Food Sciences Shiraz University of Medical Sciences Shiraz Iran; ^6^ Department of Physical Education and Sport Sciences, Science and Research Branch Islamic Azad University Tehran Iran; ^7^ Department of Clinical Nutrition, School of Nutritional Sciences and Dietetics Tehran University of Medical Sciences (TUMS) Tehran Iran; ^8^ Department of Clinical Nutrition & Dietetics, National Nutrition & Food Technology Research Institute Shahid Beheshti University of Medical Sciences Tehran Iran

**Keywords:** cranberry, DBP, meta‐analysis, randomized controlled trial, SBP

## Abstract

**Background:**

The aim of this paper, which includes a meta‐analysis, is to elucidate the effects of cranberry consumption on systolic and diastolic blood pressure based on all relevant randomized controlled trials (RCTs).

**Materials and Methods:**

A systematic literature search was performed across the ISI Web of Science, PubMed, Embase, the Cochrane Library, and Google Scholar databases, encompassing trials published until December 2024. Weighted mean differences (WMD) were calculated using random or fixed‐effects models. Between‐study heterogeneity was evaluated using Cochrane's test and the *I*² index. This study's registration number in PROSPERO is CRD420251028424.

**Results:**

A total of 1204 publications were reviewed, leading to the inclusion of 12 trials for qualitative synthesis and meta‐analysis. The pooled effect size indicated statistically nonsignificant reductions of 1.31 mmHg for systolic blood pressure (SBP) (*p* = 0.19) and 1.31 mmHg for diastolic blood pressure (DBP) (*p* = 0.12). Stratified analysis showed that the reduction in SBP was statistically significant in studies where cranberry was provided in juice form, with a duration of 8 weeks or less, involving participants with a mean age of < 50 years, and predominantly in females. Furthermore, subgroup analysis indicated a significant reduction in DBP in studies that involved both genders, lasted more than 8 weeks, included participants with a normal body mass index, and had a mean age below 50 years.

**Conclusion:**

This systematic review and meta‐analysis suggest that cranberry consumption was not effective in managing SBP and DBP.

## Graphical abstract

We examined the effects of cranberry consumption on blood pressure. Cranberry consumption has no significant effect on systolic (SBP) and diastolic blood pressure (DBP). Cranberry consumption provided in juice form, with a duration of 8 weeks or less, involving participants with a mean age of < 50 years, and predominantly in females, significantly reduced SBP. Cranberry consumption in studies that involved both genders, lasted more than 8 weeks, included participants with a normal body mass index, and had a mean age below 50 years, caused a significant reduction in SBP.

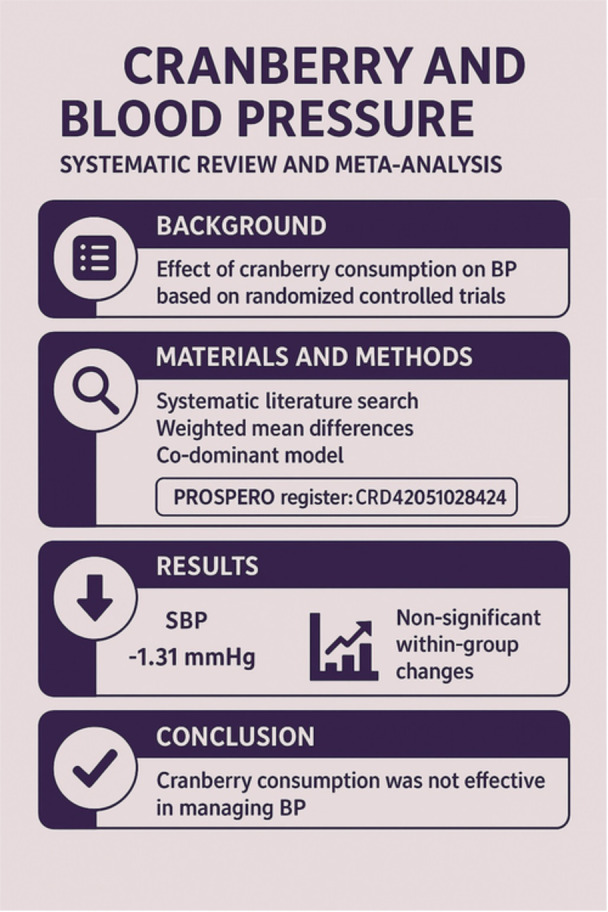

## 1 Introduction

Hypertension is defined as continuously elevated systolic blood pressure (SBP) above 140 and/or diastolic blood pressure (DBP) above 90, and it is one of the risk factors that leads to atherosclerotic cardiovascular diseases (ASCVD) and many other medical conditions [1, 2]. Based on a WHO report, hypertension is responsible for more than 10 million deaths annually [1]. The main etiology of hypertension is unclear but some risk factors that can cause hypertension include gender, genetic factors, family history, low physical activity, and unhealthy diet [1, 3, 4]. Treatment of hypertension includes lifestyle modification in the first line and along with pharmacotherapy; lifestyle modification consists of various parts including diet and complementary medicine [5].

Cranberry or Vaccinium is one of the high‐containing polyphenols among fruits and vegetables and is cultivated in the northern part of America, Canada, and some parts of Europe [6, 7]. Based on its size, small or big, the percent of components may differ but it is rich in anthocyanins (ACNs), flavonols, fiber, and different vitamins [6, 7, 8, 9]. Its potential effects include increasing plasma antioxidant capacity, reducing cardiovascular disease (CVD) risk factors, 8‐isoprostane (lipid peroxidation index), and advanced oxidized protein products (AOPPs), improving visual memory by increasing regional perfusion, prevention and treatment of bladder cancer, and being efficient in readjusting skin lipids and microbiome in women [8, 10, 11, 12, 13, 14, 15]. Some studies showed that cranberries can be effective in lowering DBP [16, 17]. On the other hand, some studies demonstrated no change in blood pressure or central systolic pressure [17, 18].

One systematic meta‐analysis, which aimed to review the effect of cranberry and blueberry supplementation on patients with CVD, included data from eight articles on cranberry; no change was seen in SDB or DBP [19].

Due to the controversies in results, small sample sizes in previous studies, and the limited number of studies, we aimed to conduct a systematic review and meta‐analysis. By incorporating subgroup analyses, a larger number of included studies, and covering all populations, we can achieve a comprehensive understanding and conclusion regarding the potential effects of cranberries on blood pressure.

## 2 Materials & Methods

### 2.1 Search Strategy

The present systematic review and meta‐analysis was performed according to the Preferred Reporting Items for Systematic Reviews and Meta‐analyses (PRISMA) [20]. The registration number for this study in PROSPERO is CRD420251028424. Electronic databases, including PubMed, Web of Science, and Scopus, were searched to include trials published up to December 2024. We used the following keywords in our non‐MeSH terms: (Cranberry OR “*Vaccinium macrocarpon”* OR “*Vaccinium microcarpum”* OR “*Vacciniumoxycoccus”*) in combination with (“Blood Pressure” OR “Pressure, Blood” OR “Diastolic Pressure” OR “Pressure, Diastolic” OR “Pulse Pressure” OR “Systolic Pressure” OR Hypertension). The reference lists of retrieved and related review studies were hand‐searched to find more relevant studies. Table S1 highlights the details of the search strategy and electronic sources used.

### 2.2 Study Inclusion and Exclusion Criteria

Original articles were selected if they had the following PICOS criteria [21]: (1) Participants: aged ≥ 18 years, (2) Intervention: Cranberry supplement or cranberry juice or cranberry extracts, (3) Comparison (placebo), (4) Outcomes: having data on SBP/DBP in the baseline and the end of the intervention, and (5) Study design: randomized controlled trials (RCTs) with either parallel or cross‐over design.

Publications were excluded if they had one of the following criteria (1) research on pregnant women, children, animals, (2) non‐controlled or non‐randomized clinical trials, (3) studies without a placebo group, (4) combined cranberry with other dietary supplements or interventions, (4) duplicated studies, (5) studies with not sufficient data for analysis, and (6) review, meta‐analysis, conference papers, and protocol.

### 2.3 Data Extraction

Two independent reviewers (L.Z.B. and M.R.A.) screened the titles and abstracts and then the full text of relevant studies by considering the above inclusion and exclusion criteria. Finally, the references of the selected studies were reviewed. Any disagreement between the reviewers were discussed and solved by the third reviewer (A.H.). We used Excel 2019 and the standard form to extract the main information from the eligible articles.

### 2.4 Quality Assessment of Meta‐Analysis

Two reviewers (L.Z.B. and M.R.A.) independently assessed the risk of bias for each RCT using the Cochrane Risk of Bias Tool for RCTs [22]. This tool contains seven domains including (1) random sequence generation (selection bias), (2) allocation concealment (selection bias), (3) blinding of participants and personnel (performance bias), (4) blinding of outcome assessment (detection bias), (5) incomplete outcome data (attrition bias), (6) selective reporting (reporting bias), and (7) other bias. For each item, the risk of bias was categorized as low risk, high risk, or unclear. Discuss with the third researcher (A.H.) was our selection to resolve any inconsistencies between the two reviewers.

### 2.5 Statistical Analyses

Data was analyzed using Stata software, version 14.0 (Stata Corp, College Station, TX). We used weighted mean differences (WMD) and 95% confidence intervals (CI) of SBP and DBP to estimate the treatment effects [23]. The following equation was used to calculate changes in standard deviation (SD): SD change = square root (SD^2^baseline + SD^2^endpoint–[2R*×*SD baseline*×*SD endpoint]) [24], correlation coefficient *R* = 0.8 [25]. When data were presented as standard error (SE), SD was estimated using this formula: SD = SEM * sqrt (*n*); *n* is the number of subjects. Inter‐study heterogeneity was evaluated using Cochran's *Q* and *I* ^2^ statistics. *I* ^2^ < 30%, *I* ^2^ = 30%–75%, and *I* ^2^ > 75% were categorized as low, moderate, and high heterogeneity, respectively [26]. As the results showed moderate heterogeneity for SBP and high heterogeneity for DPB, we applied a random‐effect model in the meta‐analysis. To assess the source of bias, we conducted subgroup analysis for the type of intervention, duration of treatment, BMI, age, and gender. The risk of publication biases was evaluated using both the visual funnel plot and Egger's test [27]. Both the visual funnel plot and Egger's test were conducted to indicate the presence of the risk of publication bias. The significance level was considered as *p* < 0.05 [28].

## 3 Results

### 3.1 Search Results

**Figure 1:**
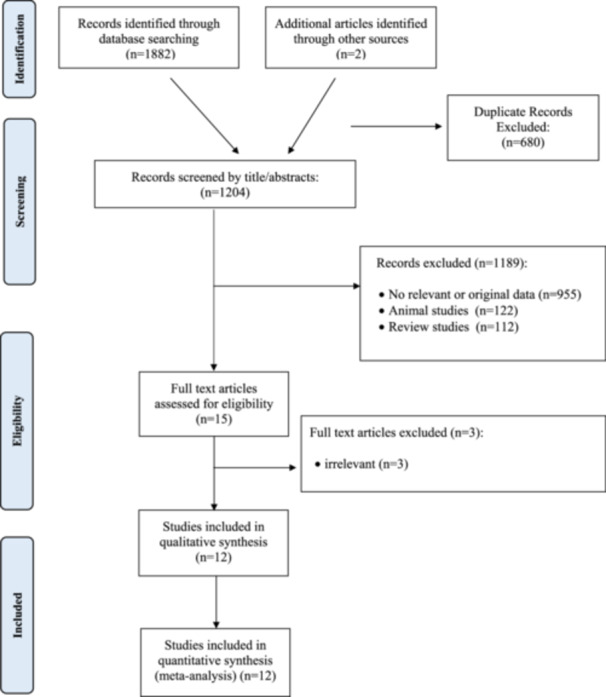
Flowchart of the number of studies identified and selected into the meta‐analysis.

Figure 1 shows the flowchart of the article selection process in this meta‐analysis. A total of 1884 articles were identified from our database searches (*n* = 1882) and the reference list of relevant articles (*n* = 2). After removing duplicates, 1204 articles were screened by title and abstracts, and 1189 articles were excluded including no relevant articles with no required data (*n* = 995), animal studies (*n* = 122), and review articles (*n* = 112). We assessed the full text of 15 eligible articles, and finally, 12 articles were included in this analysis [16, 17, 18, 29, 30, 31, 32, 33, 34, 35, 36, 37].

### 3.2 Studies Characteristics and Main Findings

Table 1 shows the main characteristics of 12 RCTs [16, 17, 18, 29, 30, 31, 32, 33, 34, 35, 36, 37] comprising 607 participants with a mean age of 49.2. Studies were published from 2008 to 2022 and conducted in the USA [16, 17, 33, 35, 36, 37], Iran [18, 31], UK [34], Taiwan [32], Canada [30], and the Czech Republic [29]. Except for two trials [17, 36] had cross‐over designs, the 10 reminded trials were parallel RCTs. Of the 12 studies, trials were performed in healthy people (*n* = 4) [16, 29, 30, 34], and other studies enrolled patients with metabolic syndrome (*n* = 2) [18, 37], type 2 diabetes (*n* = 1) [32], NAFLD (*n* = 1) [31], coronary artery disease (*n* = 1) [36], peripheral endothelial dysfunction and cardiovascular risk factors (*n* = 1) [35], elevated fasting glucose or impaired glucose tolerance (*n* = 1) [33], and elevated blood pressure [17] (*n* = 1). The three studies included only females as participants [18, 29, 37]; one study included only males [30], and the remaining eight studies involved both genders. The sample size of studies ranged from 30 [32] to 94 [31] participants. The duration of the intervention was varied from 4 to 24 weeks. The RCTs used the different types of cranberry consumption, including juice [16, 17, 29, 30, 33, 35, 36, 37] and powder [18, 31, 32, 34]. The dose of cranberry juice ranged from 400 to 1200 mg/day.

**Table 1:** Risk of bias for randomized controlled trials, assessed according to the revised Cochrane risk‐of‐bias tool for randomized trials.

Publications	Random sequence generation	Allocation concealment	Selective reporting	Blinding (participants and personnel)	Blinding (outcome assessment)	Incomplete outcome data	Other source of bias
1. Basu (2011)	L	U	L	L	L	L	L
2. Dohadwala (2011)	L	U	L	L	U	L	L
3. Eftekhari (2016)	L	L	L	L	U	L	L
4. Flammer (2012)	L	U	L	L	U	L	L
5. Flanagan (2022)	L	U	L	L	U	L	L
6. Hsia (2020)	L	L	L	L	U	L	L
7. Lee (2008)	L	U	L	L	U	L	L
8. Masnadi Shirazi (2021)	L	L	L	L	L	L	L
9. Novotny (2015)	L	U	L	L	U	L	L
10. Richter (2021)	L	L	L	L	U	L	L
11. Ruel (2013)	L	U	L	L	U	L	L
12. Valentova (2007)	L	U	L	L	U	L	L

Abbreviations: H, high risk of bias; L, low risk of bias; U, unknown.

### 3.3 Quality of the Studies and Risk of Bias

We used the Cochrane risk‐of‐bias tool for assessing each trial included in this systematic review. All 12 trials were at low risk of five domains, including random sequence generation, selective reporting, blinding participants, outcome assessment, and other sources of bias (Table 2). None of the included studies presented an overall high risk of bias in at least one domain. Four trials [17, 18, 31, 33] reported sufficient information for a domain of “selective outcome reporting” and two trials [31, 37] regarding blinding outcome assessment.

**Table 2:** Demographic characteristics of the included studies.

First author (year)	Location	Study design	Health status	Sex	Sample size	Duration (week)	Mean age (year)	Baseline BMI (kg/m^2^)	Intervention group	Comparator group	Outcome
1. Basu (2011)	USA	RCT, parallel	Metabolic syndrome	Female	31	8	52	40	480 mL Low‐calorie cranberry juice	Placebo	SBP/DBP
2. Dohadwala (2011)	USA	RCT, crossover	Coronary artery disease	Both	44	4	62	29.5	480 mL cranberry juice, double‐strength (54% juice)	Placebo	SBP/DBP
3. Eftekhari (2016)	Iran	RCT, parallel	Metabolic syndrome	Female	48	8	42	29.3	400 mg cranberry supplement	Placebo	SBP/DBP
4. Flammer (2012)	USA	RCT, parallel	Peripheral endothelial dysfunction and cardiovascular risk factors	Both	69	16	49.5	27.4	460 mL cranberry juice cocktail	Placebo	SBP/DBP
5. Flanagan (2022)	United Kingdom	RCT, parallel	Healthy	Both	60	12	65.5	25	9 g freeze‐dried cranberry powder or 100 g of fresh cranberries	Placebo	SBP/DBP
6. Hsia (2020)	USA	RCT, parallel	Elevated fasting glucose or impaired glucose tolerance	Both	35	8	47.5	36.9	450 mL cranberry juice	Placebo	SBP/DBP
7. Lee (2008)	Taiwan	RCT, parallel	Type 2 diabetes	Both	30	12	65.5	26	500 mg cranberry extract	Placebo	SBP/DBP
8. Masnadi Shirazi (2021)	Iran	RCT, parallel	NAFLD	Both	94	24	43.1	28.4	144 mg Vaccinium macrocarpon (equal to 13 g dried cranberry fruit)	Placebo	SBP/DBP
9. Novotny (2015)	USA	RCT, parallel	Healthy	Both	56	8	50	28	480 mL low‐calorie cranberry juice	Placebo	SBP/DBP
10. Richter (2021)	USA	RCT, crossover	Elevated Blood Pressure	Both	40	8	47	28.7	500 mL cranberry juice	Placebo	SBP/DBP
11. Ruel (2013)	Canada	RCT, parallel	Healthy	Male	35	4	45	28.3	500 mL low‐calorie cranberry juice cocktail	Placebo	SBP/DBP
12. Valentova (2007)	Czech Republic	RCT, parallel	Healthy	Female	65	8	21.6	21	400 and 1200 mL dried cranberry juice	Placebo	SBP/DBP

Abbreviations: BMI, body mass index; DBP, diastolic blood pressure; NAFLD, non‐alcoholic fatty liver disease; RCT, randomized controlled trial; SBP, systolic blood pressure.

### 3.4 Effect of Cranberry on SBP

**Figure 2:**
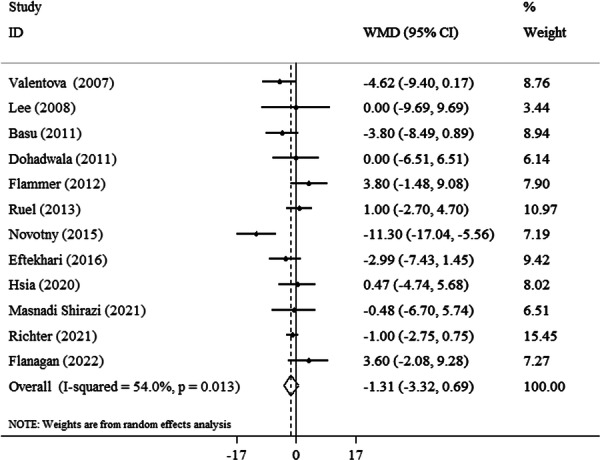
Forest plot detailing weighted mean difference and 95% confidence intervals (CIs) for the effect of cranberry consumption on SBP.

Meta‐analysis reveals cranberry administration had no significant reduction in SBP (−1.31 mmHg, 95% CI: −3.32 to 0.96, *p* = 0.19, *I* ^2^ = 54%) (Figure 2). In subgroup analysis, the effect of cranberry was significant in trials that used the juice of cranberry (−1.31 mmHg, 95% CI: −3.32 to 0.96, *I* ^2^ = 65.7%), in trials with equal or less than 8 weeks intervention (−1.75 mmHg, 95% CI: −3.01 to −0.45, *I* ^2^ = 58.8%), in patients with ≤ 50 years (−1.30 mmHg, 95% CI: −2.57 to −0.03, *I* ^2^ = 64.6%), and in females (−3.76 mmHg, 95% CI: −6.43 to −1.09, *I* ^2^ = 0.0%). No significant effect of cranberry on SBP was observed in other subgroup analyses (Table 3).

**Table 3:** Subgroup analysis of included randomized controlled trials in meta‐analysis of the effect of cranberry consumption on blood pressure.

Group	No of trials	WMD (95% CI)	*p* value	*I* ^2^ (%)	*p*‐heterogeneity	*p* for between‐subgroup heterogeneity
*SBP*						
Type of intervention						0.58
Powder	4	−0.45 (−3.35, 2.46)	0.76	6.80	0.35	
Juice	8	−1.33 (−2.60, −0.05)	0.04	65.7	0.005	
Duration (week)						0.01
≤ 8	4	−1.75 (−3.01, −0.45)	0.006	58.8	0.01	
> 8	8	2.28 (−0.83, 5.39)	0.15	0.0	0.68	
Age						0.65
≤ 50	8	−1.30 (−2.57, −0.03)	0.04	64.6	0.006	
> 50	4	−0.56 (−3.56, 2.45)	0.71	23.8	0.26	
Mean BMI						0.30
< 25	1	−4.62 (−9.40, 0.17)	0.06	—	—	
25−29.9	9	−0.84 (−2.13, 0.44)	0.20	60.2	0.01	
≥ 30	2	−1.89 (−5.37, 1.59)	0.29	29.9	0.23	
Sex						0.07
Both	8	−0.80 (−2.19, 0.59)	0.26	62.1	0.01	
Female	3	−3.76 (−6.43, −1.09)	0.006	0.0	0.88	
Male	1	1.00 (−2.70, 4.70)	0.60	—	—	
*DBP*						
Type of intervention						< 0.001
Powder	4	−0.37 (−2.08, 1.35)	0.68	0.0	0.73	
Juice	8	−1.75 (−2.72, −0.78)	< 0.001	79.8	< 0.001	
Duration (week)						0.53
≤ 8	8	−1.59 (−2.59, −0.85)	0.21	79.2	< 0.001	
> 8	4	−0.99 (−2.56, 0.57)	0.002	18.7	0.29	
Age						0.02
≤ 50	8	−1.87 (−2.81, −0.94)	< 0.001	87.4	< 0.001	
> 50	4	0.67 (−1.32, 2.66)	0.51	0.0	0.96	
Mean BMI						0.07
< 25	1	−4.57 (−8.68, −0.46)	0.03	—	—	
25−29.9	9	−1.49 (−2.39, −0.59)	0.001	75.1	< 0.001	
≥ 30	2	1.26 (−1.83, 4.34)	0.42	0.0	0.49	
Sex						0.05
Both	8	−1.81 (−2.82, −0.81)	< 0.001	76.1	< 0.001	
Female	3	−2.17 (−4.50, 0.17)	0.07	19.6	0.28	
Male	1	1.00 (−1.13, 3.13)	0.35	—	—	

Abbreviations: BMI, body mass index; DBP, diastolic blood pressure; SBP, systolic blood pressure; WMD, weight mean difference.

**Figure 3:**
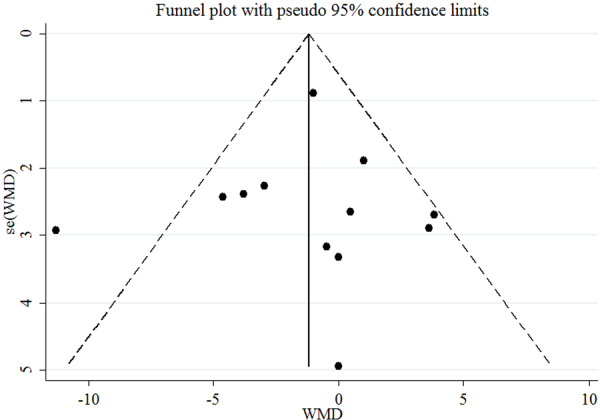
Funnel plot displaying publication bias in the studies reporting the impact of cranberry consumption on SBP.

The funnel plots for assessing the publication bias for the effects of cranberry on SBP is shown in Figure 3. The results of the Egger test revealed no publication bias for SBD (*p* = 0.86).

### 3.5 Effect of Cranberry on DBP

**Figure 4:**
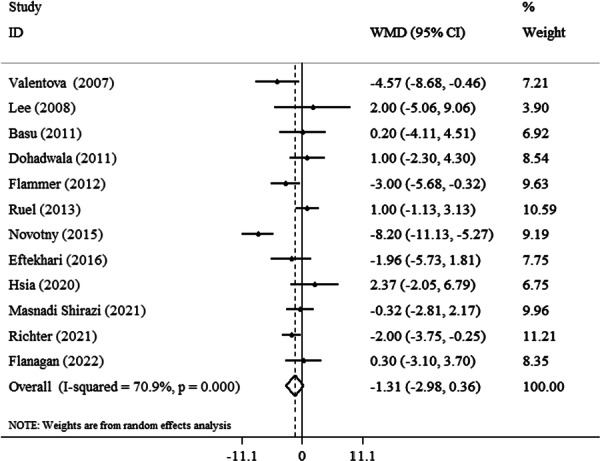
Forest plot detailing weighted mean difference and 95% confidence intervals (CIs) for the effect of cranberry consumption on DBP.

**Figure 5:**
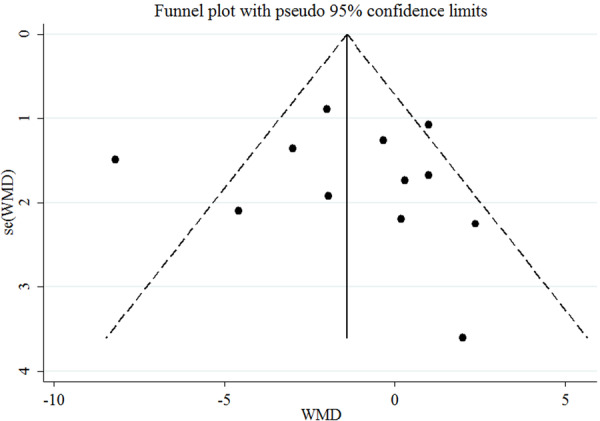
Funnel plot displaying publication bias in the studies reporting the impact of cranberry consumption on DBP.

The result of pooled effect size revealed no significant changes were detected in DBP by cranberry consumption in the intervention group in comparison with the control group (−1.31 mmHg, 95% CI: −2.98 to 0.36, *p* = 0.12, *I* ^2^ = 70.9%) (Figure 4). Subgroup analysis stratified by the dosage form and the duration of treatment indicated cranberry has a statistically significant effect on reduction of DBP in the form of juice (−1.75 mmHg, 95% CI: −2.72 to −0.78, *I* ^2^ = 79.8%) and intervention with more than 8 weeks (−0.99 mmHg, 95% CI: −2.56 to 0.57, *I* ^2^ = 18.7%). The reduction effect of cranberry on DBP was significant in participants who were ≤ 50 compared to > 50 years (−1.87 mmHg, 95% CI: −2.81 to −0.94, *I* ^2^ = 87.4%) and in studies that involved both genders compared to studies on only males or females (−1.81 mmHg, 95% CI: −2.82 to −0.81, *I* ^2^ = 79.1%) (Table 3). The funnel plots for assessing the publication bias for the effects of cranberry on DBP are shown in Figure 5. The results of the Egger test revealed no publication bias for DBP (*p* = 0.75).

## 4 Discussion

The current systematic review and meta‐analysis assessed the impact of different forms of cranberry consumption (juice and supplements) on systolic and diastolic blood pressure. The findings indicated that cranberry consumption did not lead to a significant reduction in SBP. However, subgroup analyses revealed significant effects in studies using cranberry juice, those with interventions lasting 8 weeks or less, in participants aged 50 or younger, and in females.

Regarding DBP, no significant changes were observed in the intervention group compared to the control group. However, subgroup analyses by form of cranberry consumption and treatment duration indicated that cranberry juice and interventions lasting more than 8 weeks significantly reduced DBP. Furthermore, DBP reductions were notably significant in participants aged 50 or younger and in studies that included both genders. A previous meta‐analysis examining the effects of berries on blood pressure found no significant impact on SBP and DBP in patients with cardiometabolic diseases [38]. Another review investigating the impact of cranberries on metabolic profiles found no significant effect of cranberries on blood pressure [39]. A recently published review indicated that cranberry consumption may lower SBP but does not have a significant effect on DBP [40]. While previous reviews on the effects of cranberries on blood pressure were based on a limited number of studies, our research incorporated 12 RCTs to perform a meta‐analysis, intending to obtain more reliable findings in this field.

The polyphenols found in various plant foods exhibit a range of bioactivities, including antioxidant, anti‐inflammatory, and anti‐proliferative effects, which likely contribute to the benefits observed from their consumption. Cranberries are particularly abundant in flavonoids, such as proanthocyanidins (PAC), ACNs, flavanols, and flavonols, as well as phenolic acids like benzoic, hydroxycinnamic, and ellagic acids [41, 42, 43, 44]. Some human studies have clearly demonstrated the positive effects of cranberry consumption on blood pressure [45, 46]. The blood pressure‐lowering effects of cranberries may be linked to their antioxidant components, which play a vital role in mitigating the harmful effects of reactive oxygen species (ROS), significant contributors to oxidative stress and its impact on endothelial function [44, 47]. Research involving the elderly has indicated that antioxidants can effectively manage ROS levels and stimulate antioxidant enzymes, as well as activate nitric oxide synthase (NOS) [48]. Polyphenols possess both hydrophobic and hydrophilic properties, allowing them to diffuse through biological membranes and affect intracellular signaling or biological activities, leading to the scavenging of ROS [44, 49, 50]. A recent study has identified a novel pathway in animals involving the activation of Nuclear Factor Erythroid‐2 Related Factor‐2 (Nrf2), which may enhance endothelial function and help prevent hypertension in mice exposed to Ang II‐induced conditions [49, 51]. Additionally, phenolic compounds have shown the ability to improve arterial function while promoting beneficial bacteria for health [52]. Epidemiological studies support a link between ACNs and blood pressure regulation [53, 54, 55]. Another mechanism by which cranberries affect hypertension is through enzyme regulation. For example, they enhance levels of endothelial‐derived nitric oxide (NO) by modulating the expression and activity of endothelial NO synthase (eNOS) [56]. Instantly, enhancing endothelial‐derived NO levels by regulating the expression and activity of eNOS [56, 57]. ACNs are crucial for regulating Angiotensin‐Converting Enzyme (ACE) [56, 58], endothelin‐1, and thromboxanes by inhibiting the cyclooxygenase (COX) pathway [59], and they also interact with gut microbiota to influence blood pressure [60]. Flavonoids, a type of natural phenolic compound known for their anti‐hypertensive effects, operate through four main mechanisms [61, 62, 63]: they induce vasodilation by regulating potassium (K+) and calcium (Ca^2+^) ion channels; they possess antioxidant properties that affect pro‐inflammatory pathways, leading to lower blood pressure; they promote vasorelaxation by increasing NO levels, which is essential for hypertension control and vascular homeostasis; and they counteract ROS while exhibiting anti‐radical activity in a structure‐dependent manner, with C4 and C3 hydroxyl groups enhancing their scavenging capacity.

The form of cranberry consumption appears to influence the results. Different formulations, such as juice, capsules, or whole berries, may yield varying effects on blood pressure outcomes. According to subgroup analysis, the cranberry juice consumption has potential to decrease SBP and DBP. In terms of knowledge, natural food antioxidants have different actions compared to synthetic or supplement antioxidants. Frequent consumption of antioxidants as part of a regular diet may influence the responses observed in control groups during clinical studies. However, their use as a dietary supplement to manage hypertension is not recommended due to insufficient evidence supporting their effectiveness [44, 49]. Most guidelines recommend regularly consuming a diet rich in antioxidant‐packed fruits and vegetables, as this has been shown to reduce oxidative stress and enhance vascular function [49, 64, 65]. Furthermore, literature clearly states polyphenols are the crucial antioxidants in food [44]. In addition, Stefano Vendramin's study statement ACNs intake via dietary has potentiality reduced blood pressure [56].

Another factor influencing our overall findings is the duration of treatment, as the length of the study appears to be crucial in determining the relationship between cranberry consumption and blood pressure. Research indicates that studies lasting 8 weeks or longer are necessary to observe significant effects of cranberry components on blood pressure [18, 33, 37, 66, 67, 68, 69, 70, 71]. This suggests a relationship with the long‐term modulation of blood pressure [61], primarily involving the renin−angiotensin−aldosterone system and the antidiuretic hormone (ADH) system [58], which are potential targets for the activity of cranberry components. Consequently, a positive impact on blood pressure was observed over the duration of cranberry intake, highlighting the relevance of intake time as a significant factor.

Another aspect affecting our results is age. The findings show a reduction in both SBP and DBP in studies with participants of both genders who have an average age under 50. Blood pressure tends to increase with age. Effective management and treatment of blood pressure can be achieved through exercise and diet [72, 73]. Elisabete Pinto's research points out that the rise in blood pressure with age is mainly associated with structural changes in the arteries, particularly the stiffening of large arteries [74]. Evidence suggests that the effectiveness of cranberry components in lowering blood pressure is linked to improvements in endothelial function [44, 47, 49, 51, 56, 57]. Consequently, the impact of cranberry consumption on reducing DBP in individuals over 50 can be understood in this context.

This systematic review and meta‐analysis have notable limitations as well as strengths that warrant careful consideration. One of the most significant strengths is the use of subgroup analysis. Moreover, this meta‐analysis includes a substantial number of articles compared to other meta‐analyses, thereby enhancing the generalizability of the findings. Although no significant relationship was found between cranberry consumption and systolic or diastolic blood pressure, subgroup analyses align with numerous previous studies that indicate a significant blood‐pressure‐lowering effect associated with cranberry consumption, suggesting that such an effect may indeed exist.

This article does present several limitations that need to be acknowledged. The primary limitation is the considerable heterogeneity in the data, particularly regarding the dosage of cranberry consumption, which remains unexplained in subgroup analyses related to DBP. Genetic factors may influence the effectiveness of cranberry consumption, but we could not assess this due to differences among the study populations. Additionally, there is a general lack of information regarding the baseline characteristics of the populations studied, the specific cranberry components used, dosages, intervention durations, and the varying effects on individuals along with potential synergistic interactions with other phenolics and bioactive phytochemicals, all of which are crucial for drawing broader conclusions. Furthermore, cranberry absorption and metabolism, affected by the unique microbiota enterotypes present in individuals—especially in the gastrointestinal tract and their specific responses to dietary bioactives—should also be considered.

## 5 Conclusions

In conclusion, the current meta‐analysis of 12 trials suggests that cranberries may have beneficial effects in reducing hypertension. However, the results regarding systolic and diastolic blood pressure were not significant. Stratified analysis showed that the reduction in SBP was statistically significant in studies where cranberry was provided in juice form, with a duration of 8 weeks or less, involving participants with a mean age of 50, and predominantly in females. Additionally, consumption for more than 8 weeks significantly reduced DBP in individuals under 50 with a normal BMI, regardless of gender. Further research will need to identify more precisely the clinical conditions and the characteristics of individuals for which an increased consumption of cranberry foods may be especially recommended and could potentially reduce the dose and/or the administration of antihypertensive medications. We recommend conducting additional studies to investigate other possible factors.

## Author Contributions

**Leyli Zahra Bahreyni:** writing–original draft. **Mohammad Reza Amini:** data curation, formal analysis, methodology. **Leila Sheikhi:** writing–original draft. **Ehsaneh Taheri:** methodology, writing–original draft. **Pardis Rahimi:** writing–original draft. **Mahnoush Mehrzad Samarin:** writing–original draft. **Fatemeh Sheikhhossein:** methodology, writing–original draft. **Sajjad Etesamnia:** writing–original draft. **Negin Lohrasbi:** writing–original draft. **Azita Hekmatdoost:** supervision, writing–review and editing.

## Conflicts of Interest

The authors declare no conflicts of interest.

## Supporting information

Supplementary Table 1.

## Data Availability

The data used to support the findings of this study are available from the corresponding author upon request.
