# Supplementary material for: The Effect of Cranberry Consumption on Blood Pressure: A Systematic Review and Meta‐Analysis of Randomized Controlled Trials
Source: Clin Cardiol. 2026 Apr 20;49(4):e70254. doi: 10.1002/clc.70254 (PMC13093061; doi:10.1002/clc.70254)
Supplement: Supplementary file 1 — Supplementary Table 1. [file CLC-49-e70254-s001.docx]

**Supplementary Table 1.** Search syntax.

| **Search syntax** | |
| --- | --- |
| (cranberry[tiab] OR "Vaccinium macrocarpon"[tiab] OR "Vaccinium microcarpum"[tiab] OR "Vaccinium oxycoccus"[tiab] OR "Vaccinium erythrocarpum"[tiab] OR "Vaccinium macrocarpon"[Mesh]) AND (hypertension[tiab] OR "Blood Pressure"[tiab] OR Prehypertension[tiab] OR BP[tiab] OR "Systolic blood pressure"[tiab] OR SBP[tiab] OR "Diastolic blood pressure"[tiab] OR DBP[tiab] OR cardiovascular[tiab] OR hypotensive[tiab] OR "Hypertension"[Mesh] OR "Blood Pressure"[Mesh] OR "Prehypertension"[Mesh]) AND (intervention[tiab] OR RCT[tiab] OR randomized[tiab] OR random[tiab] OR Randomly[tiab] OR Placebo[tiab] OR Assignment[tiab] OR trial[tiab] OR trials[tiab] OR randomised[tiab] OR "Methods"[Mesh] OR Cross-Over[tiab] OR "Double-Blind"[tiab] OR "Randomized Controlled Trial"[Publication Type] OR "Controlled Clinical Trial"[Publication Type] OR "Placebos"[Mesh] OR "Placebo Effect"[Mesh] OR "Clinical Trial"[Publication Type] OR "Clinical Trials as Topic"[Mesh] OR "Cross-Over Studies"[Mesh] OR "Double-Blind Method"[Mesh]) | **Pubmed** |
| ( TITLE-ABS-KEY ( cranberry ) OR TITLE-ABS-KEY (" Vaccinium macrocarpon" ) OR TITLE-ABS-KEY ( "Vaccinium microcarpum" ) OR TITLE-ABS-KEY ( "Vaccinium oxycoccus" ) OR TITLE-ABS-KEY ( "Vaccinium erythrocarpum" ) OR TITLE-ABS-KEY ( "Vaccinium macrocarpon" ) AND TITLE-ABS-KEY ( hypertension )  OR  TITLE-ABS-KEY ( "Blood Pressure" )  OR  TITLE-ABS-KEY ( prehypertension )  OR  TITLE-ABS-KEY ( bp )  OR  TITLE-ABS-KEY ( "Systolic blood pressure" )  OR  TITLE-ABS-KEY ( SBP )  OR  TITLE-ABS-KEY ( "Diastolic blood pressure" )  OR  TITLE-ABS-KEY ( DBP )  OR  TITLE-ABS-KEY ( cardiovascular )  OR  TITLE-ABS-KEY ( hypotensive ) AND TITLE-ABS-KEY ( intervention ) OR TITLE-ABS-KEY ( "controlled trial" ) OR TITLE-ABS-KEY ( randomized ) OR TITLE-ABS-KEY ( random ) OR TITLE-ABS-KEY ( randomly ) OR TITLE-ABS-KEY ( placebo ) OR TITLE-ABS-KEY ( assignment ) OR TITLE-ABS-KEY ( "clinical trial" ) OR TITLE-ABS-KEY ( trial ) OR TITLE-ABS-KEY ( randomised ) ) AND ( LIMIT-TO ( DOCTYPE , "ar" ) ) AND ( LIMIT-TO ( LANGUAGE , "English" ) ) AND ( LIMIT-TO ( SRCTYPE , "j" ) ) | **Scopus** |
